# Ropner Convalescent Home, Stockton-On-Tees

**Published:** 1898-07-23

**Authors:** C. M. Hillyer


### Editor's Letter-Box.

[Our Correspondents are reminded that prolixity is a great bar to publication, and that brevity of style and conciseness of statement greatly facilitate early insertion.]

### ROPNER CONVALESCENT HOME, STOCKTON-ON-TEES.

Sir,—A report of the Ropner Convalescent Home with a statement of accounts for 1897 has lately come into my hands, and I should like to call attention to some points in which it appears to me to be incorrect and misleading. I have written to the hon. treasurer (Mr. William Whitwell) for an explanation, and he refuses to go into the matter. He says: "The accounts, which were prepared in the usual manner, were received and approved by the Committee of Management and the subscribers at their annual meeting." There is nothing on the face of the accounts to show that they have been audited—no auditors' names appear in the report—or that there was any outside check upon the way in which they were prepared, and I myself was not present.

Before leaving the convalescent home I requested that a special committee should be called and all the accounts thoroughly gone into in my presence, and I had very good reasons for asking that this should be done. My request was, however, refused. Later on, when I was in lodgings at Middleton-on-Row, Mr. Reed (assistant treasurer) and Mr. Turnbull (assistant secretary) were sent to go into the petty cash accounts with me, and paid me a balance that was due to me for money I had advanced to the institution; beyond this, however, I had no opportunity of checking any of the accounts. At the same time I also asked that the inventory should be taken in my presence, so that everything should be handed over in proper form and due order. My request in this also was refused. I held the post of honorary matron to the institution. Notwithstanding this, I see I have been credited in the report with a sum of £28 12s. 3d., making it appear that I was in the receipt of a salary and allowance for board. This, of course, was not the case. I lived very simply, having almost entirely the same food as the patients and servants, in proof of which I may state that during the first three weeks the average cost of board a week per head was 7s. 2d., including the servants' and my own board, and there was no reason why it should have materially increased. After an attack of a sort of typhoid influenza (from which one of the servants whom I had been nursing eventually died, and which, I suppose, I contracted from the same cause), the committee, on October 11th, offered me an honorarium of £10 in consequence of having been overworked. I refused this entirely at first, but upon its being represented to me that the committee would feel hurt if I did not accept it, I received it in the spirit in which I believed it to be given. Since this, however, I find a very different construction has been put upon the whole thing by the secretary. This is the only sum I ever received that is not accounted for in my petty cash book and general accounts. There is, therefore, a sum of £18 12s. 3d. to be explained. It was not paid to me. The next entry I notice is "Servants' wages and charwoman, £44 8s." This sum is impossible. The total amount for the four months came to £27 13s. 4d. I engaged the servants at the following wages: Cook at the rate of £25 a year; two housemaids at the rate of £16 a year each, and kitchenmaid at the rate of £14 a year; and the sums of £2 1s. 8d., £1 6s. 8d., £1 6s. 8d., and £1 3s. 4d. passed through my hands to them every month while I was at the home. The charwomen who were employed to clean the house before the home opened were paid by me out of petty cash, and this is therefore entered in my petty cash accounts, which also include washing, carriage of goods, &c., &c., and is comprised in the report entry, "Housekeeping and petty cash per matron £31 12s. 4d." This also includes my own travelling expenses, which only amounted to a very small sum indeed, being simply my journey down to Middleton St. George (third-class) and a small amount spent in cabs and rail fare in London and Stockton when I was giving orders about some of the furniture, &c., for the home at the secretary's request. "Travelling expenses, £9 1s. 6d.," therefore, include no travelling expenses of my own, except maybe the cab from Darlington on my arrival and a pony cart into Darlington once when I had to fetch articles that were required immediately. It appears to me to be an enormous sum and ought to be accounted for in detail. I may mention that I paid my own journey back to town myself. "Advertising, printing, postages, £10 14s. 4d." is also a high item. My own postal expenses are down in my petty cash account, and I was provided with so inadequate a supply of official paper that it was necessary to supplement it with my own, which, of course, I paid for out of my own pocket. Under the head of receipts is an item, "Weighing machine pence, £1 5s. 8d." I put £1 6s. 6d. into the box myself from patients alone, not counting their friends and others who were weighed, and who often gave more than a penny each. With regard to omissions, there is no entry of the nurse's fees for the fortnight I had her while I was ill, which came to £4 4s., or of the caretaker during the winter months, or of the gardener and scavenger; neither is there any mention of the woman's accounts with whom I was lodging at the secretary's request (while the home was under repair) in order that I might superintend the alterations, painting, papering, cleaning, and arrival and unpacking of furniture. If these sums are entered under the heads of "matron's allowance and board" and "servants wages and charwoman," it is a misleading entry. I paid for my own lodgings and board after I left the home and was lodging in the village, but naturally expected the institution to do so while I was (much at my own inconvenience) doing more than matron's work before the home opened. It is a remarkable fact that my name is not once mentioned throughout the whole report. I may say that in undertaking the post as honorary matron I did not undertake to do nurse's work as well. This, however, I was frequently obliged to do, as can easily be imagined in a home with accommodation for forty patients. Between July 3rd and October 11th we had 159 patients in. Of these there were three epileptic cases who were constantly ill, several cases of bronchitis and bronchial asthma, several heart cases, a bad case of rectal abscess, advanced phthisis, and so on, all confined to bed from time to time, and requiring constant care and nursing. I asked for help, and the secretary refused to let me have it, and I was not given an opportunity of seeing the committee (till October 11th) for two months before I left. When I became ill on September 25th I was obliged to telegraph for a nurse on my own responsibility; my temperature was then very high, and besides influenza I was suffering frightfully from neuralgia. The nurse did not prove satisfactory, and I had to get up before I ought to have done so to see to household matters myself, as everything was being neglected and all going wrong. This, and the worry of the committee meeting, made me much worse, and then when I was again too ill to get up, the nurse was sent away and none other employed to take her place, and I had no one to look after me but the clergyman's wife (who was more or less useless, being quite untrained) and a young girl, who was the only servant I could hear of, whom I engaged to wait upon me and paid for myself. I think, sir, you will agree with me that the treatment to which I have been subjected is hardly what might be expected by anyone who voluntarily and gratuitously undertakes work for an institution. If such a state of things be possible in the case of an unpaid gentlewoman, is it not time that something be done and some steps taken to put a stop to the possibility of such injustice and to protect those matrons who are solely dependent upon their profession for their daily bread?—those who can neither afford the waste of time, the injury to health, or, worse still, the slur upon character that may thus be incurred, and who are not in a position either intellectually or financially to protect themselves.—I am, sir, yours faithfully, C. M. Hillyer.

July 5th, 1898.

